# Detection Progress of Selected Drugs in TLC

**DOI:** 10.1155/2014/732078

**Published:** 2014-01-16

**Authors:** Alina Pyka

**Affiliations:** Department of Analytical Chemistry, Faculty of Pharmacy, Medical University of Silesia, 4 Jagiellońska Street, 41-200 Sosnowiec, Poland

## Abstract

This entry describes applications of known indicators and dyes as new visualizing reagents and various visualizing systems as well as photocatalytic reactions and bioautography method for the detection of bioactive compounds including drugs and compounds isolated from herbal extracts. Broadening index, detection index, characteristics of densitometric band, modified contrast index, limit of detection, densitometric visualizing index, and linearity range of detected compounds were used for the evaluation of visualizing effects of applied visualizing reagents. It was shown that visualizing effect depends on the chemical structure of the visualizing reagent, the structure of the substance detected, and the chromatographic adsorbent applied. The usefulness of densitometry to direct detection of some drugs was also shown. Quoted papers indicate the detection progress of selected drugs investigated by thin-layer chromatography (TLC).

## 1. Introduction

Among the different chromatographic techniques, one of the most popular in biological and pharmaceutical analysis of bioactive substances and recommended by USP (United State Pharmacopia) is HPLC with respective detection system, for example, HPLC-UV. It is well known that the choice of the analytical method (including chromatographic methods) depends on the chemical and physicochemical properties of the compound being separated and also on the available apparatus and sensitivity needed for the analysis. Moreover, the total time of chromatographic analysis and its low cost are also important. In many papers concerning thin-layer chromatography (TLC) analysis of various drugs, TLC is an important method for qualitative and quantitative analysis of drugs because it indicates some advantages in comparison to HPLC and GC methods, which are listed below:

- TLC can be used in those situations when HPLC-UV and GC are not suitable, for example, absence of UV activity of examined compound (important for HPLC analysis) or when the absence of volatility (important for GC analysis) is observed;
- in comparison to HPLC in the case of TLC the high purity and high concentration of examined samples are not required. Unlike HPLC there is no danger that the samples impurities influence column damage and its separation property;
- TLC needs no expensive equipment and is easy to work in comparison to HPLC and GC;
- TLC allows a parallel separation and quantitative determination of many samples at the same time;
- it is possible to put on TLC plates a large volume of sample because the solvent excess removing during the sample is spotted on chromatographic plates.

For these purposes TLC greatly contributes to the analysis of different groups of drugs. The last decade shows that interest in TLC application in pharmaceutical analysis (e.g., control purity of drugs) has increased with improvements in TLC instrumentation such as TLC combined with densitometry or with MS and IR, respectively. If the standard compounds of drug are not available, identification of unknown drug components has to be done. Moreover, TLC combined with densitometer or MS and also detector enables the determination of drug components in a low range: pmol or fmol. For this reason TLC is used as one of the promising methods of separation and quantitative determination for these types of compounds for which GC or HPLC is not suitable. The above-mentioned arguments explain enough why the TLC-densitometry is proposed in many drug analyses as an alternative method to the pharmacopoeias methods.

Important step of thin-layer analysis of compounds is the detection of investigated substances including drugs. The separated substances by thin-layer chromatography (TLC) can be detected by the following methods: physical (individual colour of substance or fluorescence of substance in UV light); chemical (coloured reactions of separated substances with visualizing reagents); physicochemical (e.g., the application of isotopes as visualizing reagent); biological (the application of biodetectors) [1–10]. Many drugs are active in the range of UV light and can be directly detected and determined on chromatographic plate, for example, by densitometric analysis, in short or long wavelength UV light [11–39]. The visualizing reagents have the special significance to detect separated compounds on thin layers. In view of the detection mechanism of the compound, the visualizing reagents can be sorted as follows: conservative reagents, which do not destroy separated substances destructive reagents, which destroy or change the structure of separated substances. Currently, the most important field of application of thin-layer chromatography is pharmacy. The number of publications in the field of pharmacy steadily increases. It results from the fact that contemporary thin-layer chromatography is a fully instrumentalized and automated technique [1–3, 29].

The applications of many reagents for the detection of organic compounds, including drugs, after their separation on thin-layer were presented in books [1, 2] on the basis of manuscripts published up to 1992. The objectives of this review are the presentation and discussion of the significance of known indicators and dyes as new visualizing reagents and various visualizing systems as well as photocatalytic reactions and bioautography method for detection of bioactive compounds including drugs and compounds isolated from herbal extracts in TLC investigations that were described in the selected manuscripts from 1993.

## 2. Parameters Describing the Visualizing Effects

The estimation of visualizing effects of the detected compounds must be objective. Therefore, Gregorowicz and Sliwiok [39, 40] introduced the indexes for estimation of rhodamine B, new fuchsine, basic fuchsine, and crystal violet for detection of cholesterol and higher fatty acids. To compare the developing effects of the above-mentioned dyes, it was suggested [39, 40] to estimate the chromatographic spots obtained by means of indexes given bellow:

(i) detection index which indicates the ratio of the minimal number of micrograms of the substance detected to the area of the chromatographic spot in square millimeters obtained by the planimetric method:
  (1) $$\begin{array}{c} I_{\det}=\frac{m_{1}}{p_{1}}\;\left[\frac{\mu \text{g}}{{\text{mm}}^{2}}\right], \end{array}$$
  where *m*_1_ is the smallest quantity of substances detected [*μ*g] with the visualizing reagent (limit of detection) and *p*_1_ is the spot area of the substance [mm^2^] at the limit of detection of the substance;
(ii) developing index which gives the numerical value of the part of the chromatographic spot area relating to 1 *μ*g;
(iii) contrast index which represents two independent values, namely, the angle between tangents drawn to the arms of the densitometric band, formulated in the degrees, and the densitometric band height in centimeters;
(iv) durability of the spot in order to formulate the durability of chromatographic spot colors. The following relative scale was proposed: spot of minor durability, visible up to 2 hours; spot of mean durability, visible from 2 to 6 hours; and durable spot durability, visible for over 6 hours.

Today the area of chromatographic spot is determined by densitometric analysis. Therefore, Pyka et al. modified the above-mentioned indexes and also proposed new indexes which characterized the visualizing effects [41–45]. The limit of detection (detectability), detection index, broadening index, modified contrast index, densitometric visualizing index, and linearity range were determined and tested for estradiol, tocopherol, tocopherol acetate, ibuprofen, stearic acid, stearyl alcohol, and salicylanilide detected by the use of visualizing reagents. The broadening index [41, 42] was defined as

(2) $$\begin{array}{c} I_{\text{broad}}=\frac{a}{p_{2}}\times 1000\;\left[\frac{\mu \text{g}}{\text{AU}}\right], \end{array}$$

where *a* is the mass [*μ*g] of the detected substance and *p*_2_ is the spot area [AU] of *a* *μ*g of this analyzed substance.

The detection index was defined as

(3) $$\begin{array}{c} I_{\det}=\frac{m_{1}}{p_{1}}\;\left[\frac{\mu \text{g}}{\text{AU}}\right], \end{array}$$

where *m*_1_ is the smallest quantity of substance detected [**μ**g] (limit of detection) and *p*_1_ is the spot area of substance [AU] at the limit of detection of substance.

The modified contrast index [43] was defined as

(4) $$\begin{array}{c} I_{\text{contr(mod if)}}=\frac{h}{\alpha }\;\left[\frac{\text{AU}}{{}^{{}^{\circ}}}\right], \end{array}$$

where *h* is the height of the densitometric band [AU] of *a* *μ*g of analyzed substance and *α* is the angle [°] between the tangents at the inflection points to the curves of the densitometric band of substance.

The densitometric visualizing index (DVI) [44] was proposed and defined as

(5) $$\begin{array}{c} \text{DVI}=\frac{p_{2}}{m_{1}\times \alpha }\times 10^{-4}\;\left[\frac{\text{AU}}{{\mu \text{g}\;\cdot }^{{}^{\circ}}}\right], \end{array}$$

where *m*_1_ is the limit of detection of the analyzed substance [**μ**g], *p*_2_ is the spot area [AU] of *a* *μ*g of analyzed substance, and *α* is the angle [°] between the tangents at the inflection points to the curves of the densitometric band of *a* *μ*g of analyzed substance.

### 2.1. Application of New Parameters Characterizing the Visualizing Effects

New visualizing reagents, namely, gentian violet, methylene violet, methylene blue, methyl green, malachite green, and Janus blue, have been used to detect estradiol, tocopherol, tocopherol acetate, ibuprofen, stearic acid, and stearyl alcohol [41–43, 45]. Brilliant green was applied to detect salicylanilide [44]. Estradiol was chromatographed on neutral aluminum oxide 60F_254_ and neutral aluminum oxide 150F_254_ using a mixture of toluene and ethyl acetate (1 : 1, v/v) as mobile phase. Barton's reagent, rhodamine B, and sulphuric acid were administered as the comparative visualizing reagents [43]. (±)-*α*-Tocopherol and (+)-*α*-tocopherol acetate were chromatographed on silica gel 60 using toluene as mobile phase [41]. Rhodamine B and 2,2′-bipyridine-iron (III) chloride reagent were applied as the comparative visualizing reagents. Ibuprofen was chromatographed on silica gel 60F_254_ using a mixture of chloroform and methanol (50 : 1.4, v/v) as a mobile phase. Rhodamine B was used as the comparative visualizing reagent [42]. Stearic acid and stearyl alcohol were chromatographed on silica gel 60 using a mixture of n-hexane-ethyl acetate (46 : 4, v/v) as mobile phase. Rhodamine B was used as the comparative visualizing reagent [45]. Fundamental absorption band of studied drugs, color of spot of detected drugs, background color of chromatogram, limit of detection, and linearity range are presented in Table 1. Characteristics of densitometric band of detected drugs, modified contrast index, densitometric visualizing index, modified broadening index, and detection index are presented in Table 2. It was stated that the proposed modified contrast index is the objective parameter describing the applied visualizing reagents. Influence of solid support on the obtained visualizing effects was found. The angles (*α*) between the tangents at the inflection points to the curves of the densitometric peaks were more compact on neutral aluminum oxide 60F_254_ than on neutral aluminum oxide 150F_254_. This observation indicates that the utility of any particular visualizing reagent depends on the type of chromatographic support applied. For quantitative research of estradiol investigated, relatively good properties had sulphuric acid (VI), Barton's reagent, gentian violet, and methylene violet [43]. 2,2′-Bipyridine-iron (III) chloride reagent can be used only to detect (±)-*α*-tocopherol. Among all studied new visualizing reagents, methylene violet and methyl green were the best to detect (±)-*α*-tocopherol. The best detection way of (+)-*α*-tocopherol acetate was densitometric method without using a visualizing reagent, whereas among all studied new visualizing reagents, gentian violet, methyl green, and Janus blue were the best to detect (+)-*α*-tocopherol acetate. These visualizing reagents have similar detection properties of (+)-*α*-tocopherol acetate in relation to rhodamine B [41]. The best detection way of ibuprofen was densitometric method without using a visualizing reagent. Among all studied new visualizing reagents, methylene violet was the best to detect ibuprofen [42]. New visualizing reagents for detection of stearic acid and stearyl alcohol were better visualizing reagents in comparison with the universally applied rodamine B to detect the lipophilic compounds. The best visualizing reagents to quantitative determination of stearic acid were methylene blue and Janus blue. The best visualizing reagents to quantitative determination of stearyl alcohol were malachite green and Janus blue [45].

## 3. Visualizing Effects for Different Substances including Drugs

### 3.1. Detection by the Use of Visualizing Reagents and Various Visualizing Systems

#### 3.1.1. Detection of Steroids Compounds including Drugs

Huetos et al. [46] investigated the specificity of chromatographic conditions of 35 anabolic steroids and 10 other veterinary drugs. The best sensitivity of the different corticosteroids was performed with silica gel plates eluted with a mixture of chloroform and methanol in volume composition 92 : 8. Four spray reagents were compared for selectivity, sensitivity, and specificity. The best results were obtained using reagents 1 and 2. Reagent 1 was a 1 : 1 mixture of a solution of 2,4-dihydroxybenzaldehyde (25 mg) in glacial acetic acid (50 mL) and a mixture of sulfuric acid (12.5 mL) and glacial acetic acid (37.5 mL). Reagent 2 was a 1 : 1 mixture of a solution of tetrazolium blue (0.25 mg) in methanol (50 mL) and a solution of sodium hydroxide (10 mg) in water (50 mL) [46]. The plates after spraying were heated at 85–90°C for 10 min.  Table 3 shows *R*_*F*_ values, color of spots, and detection limits for the selected seven most common corticosteroids after reaction with reagents 1 and 2. Reagent 2 can be administered for sensitive screening for the presence of corticosteroids in illegal cocktails. Reagent 1 is more suitable. Reagent 1 gives more information about the corticosteroids, owing to the development of different colors, with reasonable sensitivity [46].

Stigmasterol and *β*-sitosterol (10 *μ*g and 5 *μ*g) were visualized after separation on silica gel using a mixture of toluene and ethyl acetate (7 : 3, v/v) [47]. The results obtained for the stigmasterol and *β*-sitosterol by use of the individual visualizing reagents (gentian violet, methylene violet, methyl green, malachite green, and Janus blue) depend on the method of application—spraying or dipping. Rhodamine B was used as comparative visualizing reagent. The best results of detection of stigmasterol and *β*-sitosterol were obtained by spraying the visualizing reagents (Tables 4 and 5) [47].

The alkacymetric indicators, namely, thymol blue, bromophenol blue, and bromothymol blue, were also used in the detection of different classes, including the compounds of biological significance, namely, sterols (cholesterol, stigmasterol) on silica gel 60F_254_ after separation using the n-hexane + acetone (8 : 2, v/v) mobile phase. The colors of spots were durable and sufficient contrast between spots and background was observed. The detection limits with these visualizing reagents were from 0.5 *μ*g to 2.0 *μ*g for sterols [48]. Alkaline blue, aniline blue, brilliant green, bromothymol blue, thymol blue, phenol red, helasol green, bromocresol green, brilliant cresyl blue, bromophenol blue, and neutral red were applied for visualizing cholesterol and its derivatives in adsorption thin-layer chromatography (on silica gel and aluminum oxide) and in argentation thin-layer chromatography (on silica gel impregnated with an aqueous solution of silver nitrate (5%)) [49]. Visualizing effects of detection of cholesterol derivative depend on several factors, including the chemical structure of the visualizing reagents, the structure of the detected substance, and the type of chromatographic sorbent. Cholesteryl acetate was revealed with all visualizing reagents after separation on silica gel. Cholesteryl acetanilide, cholesteryl acetate, and cholesteryl arachidonate could be determined on silica gel by use of all the reagents except bromocresol green. On neutral aluminum oxide 0.3 *μ*g cholesterol could be revealed with bromophenol blue, neutral red, and aniline blue; 0.3 **μ**g cholesteryl acetanilide could be visualized with bromophenol blue and alkaline blue; 0.3 **μ**g cholesteryl acetate could be detected with bromophenol blue, neutral red, helasol red, helasol green, and alkaline blue; 0.1 **μ**g cholesteryl arachidonate could be visualized with bromophenol blue. The worst results were obtained after argentation TLC; the compounds studied could be detected only by the use of phenol red, helasol green, bromophenol blue, neutral red, aniline blue, and alkaline blue. The best and most universal visualizing reagents for cholesterol and the derivatives investigated in adsorption and argentation TLC were aniline blue, bromophenol blue, helasol green, and alkaline blue [49].

Wardas and Jędrzejczak [50] separated the selected free and conjugated bile acids by NP-TLC. Eleven visualizing agents were applied for detection of these investigated bile acids. The best results of detection of bile acids were obtained with bromocresol blue [50]. Bile acids can be also visualized by dipping plates into phosphomolybdic acid in ethanol and then heating for 10 min at 105–120°C [1] or by spraying the plates with a 10% solution of phosphomolybdic acid in methanol and then heating for 20 min at 50–80°C [51]. Chromatographic bands of bile acids on the densitogram after the use of spray solution of phosphomolybdic acid in methanol were irregular [51]. Therefore, this way of the detection of bile acids cannot be recommended. Regular chromatographic bands of bile acids on the densitogram were obtained after the use of dipping water solution of sulphuric acid [52].

#### 3.1.2. Detection of Antiepileptic, Anticonvulsant, and Psychotropic Drugs

Phenytoin was detected by 1% aqueous mercuric nitrate reagent [1], 2% ethanolic mercuric chloride followed by 0.2% ethanolic diphenylcarbazone [1, 53], and 0.01% carbazone followed by 1% ferrous sulfate [1]. However, these reagents with phenytoin give a similar reaction to the barbiturates. Kulkarni et al. [54] reported a selective and sensitive chromogenic reagent, namely, 0.1% bromine in carbon tetrachloride followed by 0.1% o-tolidine in 0.5% acetic acid, for detection and determination of phenytoin in biological samples investigated by TLC. Phenytoin with this reagent gives blue spot on a white background of chromatogram. Color of blue spot is stable for an hour. This reagent does not give any color reaction with barbiturates. However, this reagent gives a similar color reaction with the benzodiazepines. Sensitivity for phenytoin, alprazolam, diazepam, lorazepam, nitrazepam, and oxazepam is equal to 5 **μ**g, 20 **μ**g, 30 **μ**g, 5 **μ**g, 30 **μ**g, and 5 **μ**g, respectively [54]. A single and sensitive chromogenic chlorination with the use of o-tolidine was used for detection of diazepam, phenobarbitone, and saccharin [55]. Diazepam, phenobarbitone, and saccharin solution were spotted on silica gel G plates and developed by the use of one from three mobile phases, namely, n-hexane-acetone-methanol (8 : 3 : 0.5, v/v/v), n-hexane-acetone-butanol (12 : 8 : 0.5, v/v/v), and chloroform-acetic acid (9 : 1, v/v). After development plates were dried in air and were placed for ca 5 min in a chamber containing chloride gas, excess chlorine was removed from the plates and the next plates were sprayed with o-tolidine reagent (50 mg o-tolidine in 100 mL 10% acetic acid). When a faint blue color appeared the plates were next sprayed with 1% phosphomolybdic acid for stabilization. The limits of detection for diazepam, phenobarbitone, and saccharin with o-tolidine reagent were 0.5 **μ**g per spot, 0.3 **μ**g per spot, and 0.1 **μ**g per spot, respectively [55]. Cyclodol and diprazin were separated on silica gel by the use of one from six mobile phases: toluene-acetone-ethanol-25% NH_4_OH (45 : 45 : 7.5 : 2.5, v/v/v/v), hexane-ethyl acetate (15 : 5, v/v), chloroform-heptene-25% NH_4_OH (16 : 3 : 3, v/v/v), ethyl acetate-hexane (1 : 1, v/v), acetonitrile-methanol (1 : 1, v/v) or heptene-chloroform-ethanol-25% NH_4_OH (5 : 10 : 3 : 1, v/v/v/v). Iodine vapours, UV light at 254 nm, and fresh normal plasma (FNP) can be applied as visualizing reagents for cyclodol and diprazin. FNP gives colored spot with diprazin [56].

#### 3.1.3. Detection of Antibiotics

Quintens et al. [57] described the procedure which enables the identification of thirty cephalosporins by TLC on silanized silica gel F_254_. The obtained results with seven mobile phases were reported. Results of some reactions with all cephalosporins were also presented. The following reagents were applied: (1) sulfuric acid; (2) sulfuric acid-formaldehyde (1 mL of a 37% m/m solution of formaldehyde mixed with 50 mL of concentrated sulfuric acid); (3) sulfuric acid-nitric acid (1 mL of concentrated nitric acid mixed with 99 mL of an 80% v/v solution of sulfuric acid) [57]. The reagents (1) and (2) were used by mixing the cephalosporin (2 mg) with 0.05 mL water and adding the reagent (2 mL). The reagent (3) was administered by mixing the cephalosporin (2 mg) directly with 0.25 mL of reagent (3). The details of results obtained with the color reactions were presented in Quintens paper [57]. The sulfuric acid-formaldehyde reagent (2) gives the most discriminatory colors. TLC on silanized silica gel combined with simple color reactions enables identification of all investigated cephalosporins [57].

Bacitracin is a mixture of related cyclic polypeptides produced by *Bacillus licheniformis* and *Bacillus subtilis *organisms and is used in human medicine as a polypeptide antibiotic. Bacitracin with dabsyl chloride gives a complex and can be detected by densitometric measurements at 460 nm [58].

#### 3.1.4. Detection of Essential Oil Compounds

Pyka et al. [59] applied eighteen new visualizing reagents for the visualization of nineteen standard essential oil components (hydrocarbons, aliphatic and monocyclic alcohols, phenols, ethers, ketones, and aldehydes) after chromatography on silica gel and benzene as mobile phase. The visualizing effects obtained were compared with detection of these compounds by using iodine vapor and 5% solution potassium dichromate in 40% sulfuric acid. These investigations possess quality character for the detection substances which occur in various essential oils. Quality identification was based on the ground of the *R*_*F*_ values of investigated compounds and on the color of detected compounds in TLC. The substances belonging to the same class of compounds investigated by means of adsorption thin-layer chromatography may have slight differences in *R*_*F*_ values. For example, hydrocarbons investigated by Pyka et al. [59], camphene, (R)-(+) limonene, and p-cymene; alcohols: farnesol, menthol, and borneol; phenols: eugenol and guaiacol; ethers: coumarin, cineole, and carvone; and cinnamic aldehyde, as well as camphor and (1R)-(−) fenchone, with regard to their incomplete separation by means of these techniques (small differences in *R*_*F*_ values), can be identified by means of diversified colours of spots on chromatogram. These results can be used in the practice for qualitative investigation of essential oils of plants. For example, thymol, linalool, and borneol are presented in thyme herb *Herba Thymi*. The difference in *R*_*F*_ values between thymol and linalool was notable and was equal to Δ*R*_*F*_ = 0.265 while between linalool and borneol was small and was equal to Δ*R*_*F*_ = 0.043. That is why the confirmation of appearance particularly linalool and borneol in mixture can be by diversified colours of their chromatographic spots with visualizing reagents: 0.1% aqueous HCl + 1% aqueous Ni(NO_3_)_2_, 0.1% aqueous HCl + 2% aqueous CuSO_4_, and 0.1% aqueous Na_2_CO_3_ + 2% aqueous CuSO_4_. Furthermore, linalool and geraniol are the main components of coriander fruit (*Fructus Coriandri*). The difference in *R*_*F*_ values between these compounds was equal to 0.085. The different colours of chromatographic spots of linalool and geraniol were obtained with visualizing reagents 0.1% aqueous HCl + 1% aqueous Ni(NO_3_)_2_, 0.1% aqueous HCl + 2% aqueous CuSO_4_, 0.1% aqueous HCl + 2% aqueous CuSO_4_ + saturated aqueous solution of variamine blue hydrochloride, 0.1% aqueous HCl + saturated aqueous solution of variamine blue hydrochloride, saturated aqueous solution of variamine blue hydrochloride, 0.1% aqueous Na_2_CO_3_ + saturated aqueous solution of variamine blue hydrochloride, and 5% solution of K_2_Cr_2_O_7_ in 40% H_2_SO_4_. Fennel oil (*Fructus Foeniculi*) contains, among others, *trans*-anethole, fenchone, and limonene. Limonene was visualized only by means of visualizing reagent 0.1% aqueous HCl + 2% aqueous CuSO_4_ + saturated aqueous solution of variamine blue hydrochloride, whilst this agent does not give the reaction with fenchone and *trans*-anethole. Only the visualizing reagent 0.1% aqueous Na_2_CO_3_ gives different colour of chromatographic spots with fenchone and *trans*-anethole. Anise oil (*Oleum Anisi*) contains, among others, *trans*-anethole, fenchone, and p-cymene. p-Cymene and *trans*-anethole give chromatographic bands with various colours with visualizing reagent: 0.1% aqueous Na_2_CO_3_ + 1% aqueous Ni(NO_3_)_2_ + saturated aqueous solution of variamine blue hydrochloride, whilst fenchone could not be detected by means of this reagent. To detect fenchone it is necessary to make a second chromatographic analysis and to apply, for example, visualizing reagent: saturated aqueous solution of variamine blue hydrochloride for fenchone detection. This reagent does not detect *trans*-anethole but with p-cymene gives band with orange-yellow colour whilst the spot has light grey colour with fenchone. The results can be also applied for quick estimation of purity of some compounds obtained synthetically. The natural vanillin is present in vanilla fruits (*Vanilla planifolia*) but synthetically can be obtained from eugenol. Vanillin (*R*_*F*_ = 0.138) and eugenol (*R*_*F*_ = 0.364) can be analysed qualitatively on chromatogram and the presence of eugenol can also be checked in the product. The diversified colours of spots of these compounds with visualizing reagents: 0.1% aqueous HCl + 1% aqueous Ni(NO_3_)_2_ + saturated aqueous solution of variamine blue hydrochloride, 0.1% aqueous HCl + 2% aqueous CuSO_4_ + saturated aqueous solution of variamine blue hydrochloride, 0.1% aqueous HCl + saturated aqueous solution of variamine blue hydrochloride, saturated aqueous solution of variamine blue hydrochloride, 0.1% aqueous Na_2_CO_3_ + 2% aqueous CuSO_4_ + saturated aqueous solution of variamine blue hydrochloride, 0.1% aqueous Na_2_CO_3_ + saturated aqueous solution of variamine blue hydrochloride, 0.1% aqueous Na_2_CO_3_ + 1% aqueous Ni(NO_3_)_2_ + saturated aqueous solution of variamine blue hydrochloride, and 5% solution of K_2_Cr_2_O_7_ in 40% H_2_SO_4_, can be useful for interpretation of the results obtained [59].

#### 3.1.5. Detection of Quinones

Quinones are a class of aromatic yellow compounds that are biologically important as coenzymes or acceptors or vitamins. Kocjan [60] investigated eleven new visualizing reagents for the detection of five quinones. Quinones were separated on silica gel 60 using a mixture of dichloromethane-n-hexane (8 : 2, v/v) as mobile phase. Variamine blue hydrochloride, hydrochloric acid, dithizone, 8-hydroxyquinoline, and different salts [Ni(NO_3_)_2_, Na_2_CO_3_, Co(NO_3_)_2_] were used to prepare the solutions of visualizing reagents. The *R*_*F*_ values and different colors of spots obtained for quinones enabled unequivocal identification of all quinones. The detection limit of detected quinones was from 0.1 **μ**g to 5 **μ**g [60].

#### 3.1.6. Detection of Phenolic Drugs and Flavonoids

Wardas et al. [61] examined alkacymetric indicators for detection of adrenaline, dopamine, phenylephrine, metaraminol, fenoterol, and bithionol after TLC separation on silica gel, polyamide 11F_254_, and a mixture of silica gel and kieselguhr by using glacial acetic acid-n-butanol-water (1 + 4 + 1, v/v) as mobile phase. They investigated the following indicators as visualizing reagents: phenol red, thymol blue, bromothymol blue, bromophenol blue, cresol green, erythrosine B, eriochrome black T, brilliant cresyl blue, eosin yellow, titan yellow, and helasol green dissolved in 5% NaOH, as well as bromocresol green, aniline blue, alkaline blue, brilliant cresyl blue, and brilliant green dissolved in water. Additionally, dimethyl yellow and thymolphthalein dissolved in methanol were applied, but before used, the chromatographic plate was sprayed with 5% NaOH. In each case the concentration of the solutions was 0.5 mg/mL. The visualizing effects were characterized by the detectability index, which was defined by Gregorowicz and Sliwiok [39, 40] using (1). Characteristics of the visualizing effect for adrenaline, dopamine, phenylephrine, metaraminol, fenoterol, and bithionol with the best visualizing reagents investigated on Kieselgel 60F_254_, Kieselgel 60/kieselguhr F_254_, and Polyamide 11F_254_ are listed in Table 6. However, phenylephrine and bithionol were not detected on polyamide 11F_254_ with none of the studied visualizing reagents [61]. Pyka et al. [62] detected silica gel 60 (E. Merck, #1.05721) following phenolic drugs bamethane, ethamivan, hexachlorophene, salicylanilide, pyrocatechine, thymol, pentazocine, phloroglucinol, eugenol, niclosamide, terbutaline, methyldopa, and norepinephrine. Plates with methyldopa, norepinephrine, terbutaline, bamethane, and ethamivan were developed with a mixture of glacial acetic acid-n-butanol-water (1 : 4 : 1, v/v) as mobile phase. Plates with phloroglucinol, pentazocine, hexachlorophene, pyrocatechine, niclosamide, salicylanilide, and thymol were developed with a mixture of chloroform-methanol (9 : 1, v/v). The plate with eugenol was developed with benzene as mobile phase. Thirteen visualizing reagents were used to detect the above-mentioned phenolic drugs. Alkaline blue, aniline blue, neutral red, and brilliant green were used as 50 mg/100 mL solutions in water. Bromophenol blue, bromothymol blue, brilliant cresyl blue, thymol blue, phenol red, bromocresol green, and helasol green were applied as 50 mg/100 mL solutions in 2% aqueous sodium hydroxide solution. Bromophenol blue solution was prepared directly before use. Plates were evaluated after spraying and heated at 100°C for 15 min. By the means of the following visualizing reagents: alkaline blue, aniline blue, bromophenol blue, bromothymol blue, alkaline solution of brilliant cresyl blue, bromocresol green, helasol green, and aqueous solution of brilliant cresyl blue, it is possible to detect all of the drugs investigated in the amount of 100 **μ**g. The results with the best visualizing reagents are also presented in Table 6.

Smolarz et al. [63] visualized phenolic acid in the petioles of *Rheum undulatum *and *Rheum rhaponticum*. Separation and identification of phenolic acids were performed on cellulose plates and by using the mobile phases: benzene-methanol-acetic acid-acetonitrile (80 : 10 : 5 : 5, v/v/v/v) in the first direction and sodium formate-formic acid-water (10 : 1 : 200, w/v/v) in the second direction. After drying the chromatograms were sprayed with diazotized sulfanilic acid in 20% sodium carbonate solution or with 2% aqueous ferric chloride. The limits of detection for gallic, protocatechuic, caffeic, p-hydroxybenzoic, p-coumaric, syringic, vanillic, and ferulic acids were equal to 13 ng, 10 ng, 12 ng, 16 ng, 60 ng, 60 ng, 11 ng, 10 ng, and 64 ng, respectively [63].

Phenolic acids and flavonoids were also detected in some Croatian *Stachys Taxa*. Analysis was performed on silica gel 60F_254_ HPTLC plates using ethyl acetate-acetic acid-formic acid-water (100 : 11 : 11 : 26, v/v/v/v) as mobile phase. After drying the plates were sprayed with the natural products reagent polyethylene glycol. After spraying, chromatograms were observed at 254 nm and 366 nm. Fluorescence colors of phenolic acid and flavonoid standards were intense light blue (for caffeic acid, chlorogenic acid), dark orange (for hyperoside, isoquercitrin, luteolin, luteolin 7-O-glucoside, rutin, quercetin, and quercitrin), and light orange for vitexin [64]. Selected flavonoids (rutin, narcissin, nicotiflorin, and isoquercitrin) were isolated and identified in the extract from *Caragana spinosa *[65]. TLC was performed on silica gel plates using ethyl acetate-1,2-dichloroethane-acetic acid-85% formic acid-water (10 : 2.5 : 1 : 1 : 0.8, v/v/v/v/v) as mobile phase. First, the being development distance was 6 cm. Next, plates were dried and developed using the same conditions. After dried the investigated flavonoids were detected using visualizing reagent (0.5% 2-aminoethyl diphenylborinate solution in ethyl acetate/5% polyethylene glycol solution in 1,2-dichloroethane) [65, 66]. The detection limits were 44.00, 44.21, 27.28, and 21.29 ng for rutin, narcissin, nicotiflorin, and isoquercitrin, respectively.

Głowniak et al. [67] analyzed phenolic compounds in the flowers of *Lavatera trimestris *L. (Malvaceae). Analysis was performed on silica gel 60 using different mobile phases. Caffeic, p-coumaric, ferulic, protocatechuic, gentisic, chlorogenic, and gallic acids in UV light give blue, light blue, blue, violet, blue, blue, and violet spots, respectively. Caffeic, p-coumaric, ferulic, protocatechuic, gentisic, chlorogenic, and gallic acids in UV light after treatment with ammonia vapor give blue, blue, blue, violet, yellow, yellow-green, and violet spots, respectively. Caffeic, p-coumaric, ferulic, protocatechuic, gentisic, chlorogenic, isovanillic, gallic, syringic, vanillic, and p-hydroxybenzoic acids after spraying with diazotized sulfanilic acid in 20% sodium carbonate solution give brown, red, violet, brown, gray, light brow, orange, light brown, dark red, orange, and yellow spots, respectively. Caffeic, p-coumaric, ferulic, protocatechuic, gentisic, chlorogenic, gallic, syringic, vanillic, and p-hydroxybenzoic acids after spraying with diazotized p-nitroaniline give brown, brown-blue, blue, red-brown, gray-green, lemon, green-red, blue, violet, and red spots, respectively. Caffeic, ferulic, protocatechuic, gentisic, chlorogenic, isovanillic, gallic, and vanillic acids after spraying with a 2% aqueous solution of ferric chloride give brown-green red, light-orange, brown-blue, navy blue, gray-green, gray, and brown spots, respectively [67].

Bhujbal and Sandhya [68] investigated the extracts from two commercial formulations of *Vidangarista*. Analysis was performed on silica gel 60F_254_ plates. The presence of gallic acid and conessine in extracts was observed using ethyl acetate-toluene-methanol-formic acid (3 : 3 : 0.3 : 0.8, v/v/v/v) and toluene-ethyl acetate-diethyl amine (6.5 : 2.5 : 1.0, v/v/v) as mobile phases, respectively. After development, gallic acid and conessine on chromatographic plate were visualized using Dragendorff's reagent. Densitometric analysis was performed at *λ*_max_ 280 nm for gallic acid and at *λ*_max_ 520 nm for conessine.

#### 3.1.7. Detection of Fatty Acids, Fatty Alcohols, Amide of Fatty Acids, and Esters of Fatty Acids

The fatty acids, fatty alcohols, and esters of higher fatty acids have great biochemical, medical, and pharmaceutical significance. The alkacymetric indicators, namely, thymol blue, bromophenol blue, and bromothymol blue, were also used for the detection of different classes, including the compounds with biological significance, namely, acids (myristic acid, palmitic acid, and stearic acid), glycerides (glycerol trioleate, glycerol tripalmitate), alcohols (oleyl alcohol, stearyl alcohol), and amides (stearamide, palmitamide) on silica gel 60F_254_ after separation using the n-hexane + acetone (8 : 2, v/v) as mobile phase. The colors of spots were durable and sufficient contrast between spots and background was observed. The detection limits with these visualizing reagents were from 0.5 **μ**g to 1.0 **μ**g for acids, from 1.0 **μ**g to 2.0 **μ**g for glycerides, from 1.0 **μ**g to 2.0 **μ**g for alcohols, and 1.0 **μ**g for amides [48]. Aniline blue, alkaline blue bromothymol blue thymol blue, bromophenol blue, phenol red, helasol green, bromocresol green, and brilliant cresyl blue were also applied to the detection of unsaturated fatty acids (*cis-*6-octadecenoic, *cis-*9-octadecenoic, *cis-*11-octadecenoic, *cis-*12-octadecenoic, *cis-*13-octadecenoic, *trans-*9-octadecenoic, *trans-*11-octadecenoic, *cis-*6,9,12-octadecatrienoic, and *cis-*9,12,15-octadecatrienoic) chromatographed on silica gel 60F_254_ and on mixture of silica gel and kieselguhr F_254_ [69]. The best detectability (2 **μ**g or 3 **μ**g) of unsaturated fatty acids was obtained with aniline blue, alkaline blue bromothymol blue thymol blue, and bromophenol blue [69]. Niestrój et al. [70] separated palmitic acid, *α*-hydroxypalmitic acid, stearic acid, and 12-hydroxystearic acid by normal phase thin-layer chromatography (NP-TLC) using a mixture of n-hexane and acetone in volume composition 7 : 3 (v/v) as mobile phase. This group of compounds is not active in the range UV light. Therefore suitable visualizing reagent was applied, namely, aqueous solution of rhodamine B, which activates the detected chromatographic band [70]. The linearity range of stearic acid using rhodamine B has been established to be from 2 **μ**g to 20 **μ**g [70]. However, short-chain fatty acids from ethanoic to octanoic were chromatographed on silica gel with n-hexane-acetone (4 : 1, v/v) and acetone-water-chloroform-ethanol-aqueous ammonia (30 : 1 : 3 : 5 : 1, v/v) as mobile phases [71]. New visualizing reagents (aniline blue, alkaline blue, neutral red, brilliant green, bromothymol blue, brilliant cresyl blue, thymol blue, bromophenol blue, phenol red, bromocresol green, and helasol green) were used for the detection of the free fatty acids and their ammonium salts. The results obtained for the free fatty acids and their ammonium salts by the use of the individual visualizing reagents depend on method of application—spraying or dipping. Dipping of chromatographic plates into solutions of the visualizing reagents leads to chromatographic spots of the fatty acids with better contrast than the spots obtained by direct spraying of the plates. Generally, better visualization was achieved with ammonium salt of the fatty acids than with the free acids. Of the reagents described in the scientific literature free fatty acids from ethanoic to octanoic can be visualized only by the use of bromocresol green, bromophenol blue, potassium permanganate, and methyl red. Among the visualizing reagents investigated, dipping of chromatographic plates into an aqueous solution of alkaline blue enables the detection of the free acids from propanoic to octanoic. The ammonium salts of fatty acids from ethanoic to octanoic can be detected by spraying or dipping using the visualizing reagents investigated except bromophenol blue [71]. Eighteen new reagents (alkaline blue, aniline blue, brilliant green, neutral red, bromothymol blue, thymol blue, phenol red, helasol green, bromocresol green, brilliant cresyl blue, bromophenol blue, eriochrome black, erythrosin B, eosin yellow, spands, thymolphthalein, and Congo red) were applied for visualizing eight esters of higher fatty acids after chromatography on silica gel, on a mixture of silica gel and kieselguhr and on neutral aluminum oxide [72]. For all the esters investigated the best detectability was obtained on the mixture of silica gel 60 and kieselguhr F_254_; it was worse on silica gel 60F_254_ and the worst on neutral aluminum oxide. Bromophenol blue was the best and most universal visualizing reagent for all the esters investigated on all the chromatographic supports [72].

#### 3.1.8. Detection of Quinolones

The quinolones were detected by the Dragendorff, Forrest, and Folin-Ciocalteu reagents as well as iron (III) chloride in hydrochloric acid, iodic reagent, phosphomolybdic acid in sulphuric (VI) acid [73, 74], and terbium (III) and europium (III) ions [75]. Wardas et al. [76] also visualized quinolones using different indicators (eosin yellow, thymol blue, bromothymol blue, thymolphthalein, helasol green, spands, and titan yellow). For all the quinolone compounds studied, whether chromatographed on silica gel or aluminum oxide, the best detectability was obtained with helasol green [76]. Bober [77] showed the possibility of usefulness of the visualizing reagents such as Janus blue, methylene violet, gentian violet, methyl green, cresol red, Rhodamine B, malachite green, methylene blue, eosin yellow, and metanil yellow in the detection of selected quinolones and fluoroquinolones. The cinoxacin, pipemidic acid, ofloxacin, and pefloxacin were separated by thin-layer chromatography (TLC) and high performance thin-layer chromatography (HPTLC) on silica gel 60 using acetonitrile + water + acetic acid (6 : 40 : 4, v/v/v) as mobile phase. The visualizing effects were evaluated 3 times, namely, directly after dipping in solutions of visualizing reagents, after drying at a room temperature during 24 hours, and after dipping in solutions of visualizing reagents and drying at 120°C during 10 minutes. Cresol red was the best visualizing reagent for the detection of ofloxacin and pefloxacin. Janus blue was the best visualizing reagent for the detection of cinoxacin and pipemidic acid. The detection limits for ofloxacin, pefloxacin, cinoxacin, and pipemidic acid were 0.50, 0.75, 0.10, and 0.10 **μ**g, respectively [77]. However, ciprofloxacin HCl in coated tablets can be also directly obtained on silica gel plate 60F_254_ after scanning in absorbency mode at 278 nm [22].

#### 3.1.9. Detection of Hypnotic Compounds

Zolpidem and zopiclone are hypnotic reagents and can be used as potential drugs. Pushpalatha et al. [78] applied 0.5% solution of chloranilic acid in 1,4-dioxane for the detection of zolpidem and zopiclone on silica gel 60. Colored complex spots of zolpidem and zopiclone with chloranilic acid are formed in nonaqueous medium. Zolpidem with chloranilic acid gives dark pink spot with sensitivity of method equal to 2 **μ**g. Zopiclone with chloranilic acid gives light pink spot with sensitivity of method equal to 5 **μ**g.

#### 3.1.10. Detection of Saponins

Saponins have anti-inflammatory, antifungal, hemolytic, cytotoxicity, molluscicidal, antitumor, antiviral, and immunomodulatory properties. Saponins were detected on silica gel 60 TLC and HPTLC plates with 10% sulfuric acid in ethanol or anisaldehyde-sulfuric acid reagent and heated at 110°C in an oven for 5 min and then evaluated in visible and UV light at *λ* = 365 nm. Saponins can be also visualized by using blood reagent [79].

#### 3.1.11. Detection of Glucosamine

Glucosamine is an amino sugar, a prominent precursor in the biochemical synthesis of glycosylated proteins and lipids as well as dietary supplements used by adults. On heating the amino plate the glucosamine reacts to form a compound which strongly absorbs light between 305 nm and 330 nm, with weak fluorescence. The detection limit in absorption mode is equal to 25 ng per spot [80]. Ninhydrin has usually been applied, in combination with acetic acid, pyridine, and collodine, for the detection of glucosamine. Ammonium bisulfate, anisaldehyde, and Elson-Morgan reagents were also used for the detection of glucosamine. The detection limit of glucosamine with ninhydrin is equal to 1000 ng per spot [80].

Esters et al. [81] separated glucosamine from the plant extracts on a silica gel 60F_254_ HPTLC plates using 2-propanol-ethyl acetate-ammonia solution (8%) in volume composition 10 : 10 : 10 as mobile phase. For visualization, the plate was dipped into a modified anisaldehyde (detection reagent solution consisting of diethyl ether-glacial acetic acid-anisaldehyde-sulphuric acid in volume composition (136 : 91 : 1 : 20)) for exactly 2 s and heated at 120°C for 30 min in a drying oven. Glucosamine gives brownish-red chromatographic spots. Densitometric quantification was performed at 415 nm [81].

#### 3.1.12. Detection of Hypoglycemic Drug

Pioglitazone is a hypoglycemic (antihyperglycemic, antidiabetic) drug used to improve glucose control in adults over the age of 18 with type 2 diabetes. Mohamed et al. [82] elaborated new sensitive and selective high performance thin-layer chromatography (HPTLC) coupled with densitometry for determination of pioglitazone hydrochloride in pharmaceutical formulations. Analysis was performed on silica gel 60 with methanol-chloroform (10 : 1, v/v) as mobile phase. Chromogenic reagent as visualizing reagent, namely, o-phthalaldehyde, gives the pink colored product with pioglitazone hydrochloride on chromatographic plate.

#### 3.1.13. Detection of Selected Compounds in Extract from Different Plants

Nowak et al. [83] investigated the extracts from Asteraceae family (*Centaurea bella, Dugaldia hoopesii, Inula aschersoniana, Serratula wolffii, Stizolophus balsamita, *and* Zoegea baldschuanica*) using silica gel 60 plates. Three mobile phases were used. Methylene chloride-acetone mobile phases in different volume compositions (8 : 1, 7 : 1, 5 : 1, 3 : 1, 2 : 1, 1 : 1) were used for the separation of sesquiterpenes. However, methylene chloride-acetone mobile phase in volume composition 1 : 2 and methylene chloride-methanol in volume composition 5 : 1 were applied for the separation of ecdysones. Sesquiterpenes and ecdysones were detected on thin-layer using anisaldehyde reagent (anisaldehyde 0.5 mL, acetic acid 10.0 mL, methanol 85 mL, and sulphuric acid 4.5 mL) and heated at 103°C for 3 min. The spots of isolated compounds have different colors, namely, mauve (for stizolin), violet (for 9*α*-hydroxyparthenolide), pink (for parthenolide and cynaropicrin), orange (for hymenoratin B), yellow for hymenoratin B 2-O-*β*-D-(6′-O-acetyl)-glucopyranoside, dark violet (for kandavanolide), dark blue (for cebellin O), cherry-red (for limonin A), grey (for acetylhymenograndin), and blue (for polypodine B) [83]. Anisaldehyde reagent (1.5 mL p-anisaldehyde, 2.5 mL sulphuric acid, and 1 mL acetic acid in 37 mL ethanol) and/or Natural Product Reagent (1 g diphenylborinic acid aminoethyl ester in 200 mL of ethyl acetate) were applied to the identification of thin-layer of the compounds from the extracts of the constituent plants (*Urtica dioica *L.*, Arctostaphylos uva-ursi *L.*, Helichrysum italicum Roth, Ribes nigrum *L.*, Citrus limon *L.*, Centaurium erythraea *Rafn.*, Melissa officinalis *L., and* Arctium lappa *L.) [84].

Podolak et al. [85] elaborated HPTLC-densitometric method for quantitative determination of triterpene saponins in various parts of eight *Lysimachia *L.species (roots, stems, leaves, fruits, and flowers of *Lysimachia nemorum *L.*, Lysimachia nummularia *L.*, Lysimachia vulgaris *L.*, Lysimachia punctate *L.*, Lysimachia thyrsiflora *L.*, Lysimachia ephemerum *L.*, Lysimachia ciliate *L., and* Lysimachia clethroides*). Plates were developed with two mobile phases. First mobile phase was chloroform-methanol-water (8 : 7 : 1, v/v) for *L. ciliata, L. punctata,* and *L. nemorum*. Second mobile phase was n-butanol-acetic acid-water (6 : 1 : 3, v/v/v) for *L. ephemerum, L. vulgaris, L. thyrsiflora,* and *L. nummularia*. Plates with saponins after the separation were detected using 25% solution of sulphuric acid in methanol and heated at 105°C for 5 min. Saponin spots showed violet color. Quantitative determination of saponins was performed by scanning densitometry at 545 nm.

Yadav and Gupta [86] analyzed *Premna integrifolia*, which is general component of herbal formulation “Dashmool.” 10-O-*trans*-p-Coumaroylcatalpol, 4′′-hydroxy-E-globularinin, and premnosidic acid are major iridoid glycosides present in *Premna integrifolia*. These compounds were separated on silica gel 60F_254_ using ethyl acetate-methanol-water-acetic acid (80 : 12 : 6 : 2, v/v/v/v) as mobile phase. After development, the plates were dried and dipped into vanillin-sulphuric acid reagent (5 g vanillin + 475 mL ethanol + 25 mL sulphuric acid) and next were heated for 3 min at 110°C. Quantitative determination of investigated compounds was performed by scanning densitometry at 510 nm.

Maurya and Srivastava [87] elaborated new HPTLC-densitometric method for the simultaneous quantitation of four bioactive markers: ursolic acid, betulinic acid, *β*-sitosterol, and lupeol, in the stem and root barks of *Alstonia scholaris*. Analysis was performed on silica gel 60F_254_ plates using chloroform-methanol (99 : 1, v/v) as mobile phase. Ursolic acid, betulinic acid, *β*-sitosterol, and lupeol were visualized by vanillin-sulphuric acid reagent. Quantitative determination of ursolic acid, betulinic acid, *β*-sitosterol, and lupeol was performed by scanning densitometry at 680 nm.

Alkaloids occurring in leaves of *Lobelia cardinalis* (Campanulaceae) on thin-layer were visualized with Dragendorff's reagent [88].

#### 3.1.14. Detection of Vitamins

Hydrophobic and hydrophilic vitamins were detected by many visualizing reagents, which were described in review [29].

### 3.2. Detection by Photocatalysis of Different Drugs

Makowski et al. [89] applied the photocatalytic reactions to visualize drugs (antibiotics, antirheumatic, anti-inflammatory, analgesic, antitussive, broncholytic, spasmolytic, anesthetic, hypnotic, sympathomimetic, and vitamin). The drugs were separated on silica gel using a mixture of butanol, anhydrous acetic, and water (6 : 2 : 2, v/v/v) as mobile phase. After development and air-drying, plates were sprayed with the four visualizing reagents, namely, (a) 10 mL potassium permanganate with 0.25 g titanium dioxide powder, and after spraying, plates were illuminated for 10 min; (b) 10 mL potassium iodide with 0.25 g titanium dioxide powder, and after spraying plates were illuminated for 5 min and next were sprayed again with 2% aqueous starch solution; (c) 10 mL potassium bromide with 0.25 g titanium dioxide powder; (d) 10 mL potassium chloride with 0.25 g titanium dioxide powder. After spraying with (c) and (d) reagents the plates were illuminated for 10 min. Next these plates were sprayed with silver nitrate (0.1 mol·L^−1^) and illuminated again for 3 min. The most sensitive detections were obtained with a reagent containing titanium dioxide powder in potassium bromide solution. The detection limits with this reagent were 0.5 *μ*g for benzyl-penicillin procaine, 0.2 *μ*g for benzyl-penicillin potassium, 0.2 *μ*g for penicillic acid, 0.5 *μ*g for tetracycline hydrochloride, 0.5 *μ*g for oxytetracycline hydrochloride, 0.5 *μ*g for chlortetracycline hydrochloride, 0.2 *μ*g for penicillamine, 0.3 **μ**g for metamizole sodium, 0.2 *μ*g for aminophenazone, 0.3 *μ*g for salicylamide, 3 *μ*g for codeine phosphate, 1 *μ*g for aminophylline, 0.7 *μ*g for papaverine hydrochloride, 10 *μ*g for phenazone, 0.2 *μ*g for procaine hydrochloride, 0.2 *μ*g for phenobarbital, 0.5 *μ*g for ephedrine hydrochloride, and 0.5 *μ*g for ascorbic acid [89].

### 3.3. Microbiological Detection (Bioautography Detection)

Sensitive, selective, and rapid methods are presented for detecting inhibition of microbial growth by various compounds. Bioautography is a special method of the detection and is suitable for investigating antimicrobial activity by the application of thin-layer chromatography [90]. Bioautography can be realized in three versions, namely, contact, immersion, and direct [91]. Many antibacterial and antifungal compounds can be detected by bioautography method [90–92]. Many applications of bioautography detection are presented and discussed in review articles [90–92].

Amphotericin B [93] and Brazilian medicinal and fruit bearing plants [94] were visualized with *Candida albicans. *Shiitake mushroom was detected with *Micrococcus luteus* [95]. Two-dimensional thin-layer chromatography (2D-TLC) bioautography with *C. fragariae *was used for the detection of agricultural fungicides and antifungal drugs [96]. Vitamin B_12_ can be detected using *Escherichia coli* [97]. *Pseudomonas savastanoi *pv.* phaseolicola* was applied for the detection of aflatoxin B_1_, aflatoxin B_2_, aflatoxin G_1_, aflatoxin G_2_ [98–100], *trans*-resveratrol [101–104], antibacterial compounds in both red and white wine extracts [104], and *Chelidonium majus* L. alkaloids [105]. Doxycycline [106], flumequine [106, 107], cefacetrile [108], enrofloxacin [109, 110], ciprofloxacin [109, 110], and *Hypericum brasiliense *polyphenols [111] can be detected with *Bacillus subtilis*. Essential oils and extracts from different plants can be visualized with *Bacillus subtilis, B. cereus, Vibrio fischeri, C. sphaerospermum* (Penzig), *C. cladosporioides* (Fresen.) de Vries, *Staphylococcus aureus*, *Micrococcus luteus*, *Staphylococcus epidermidis*, *Escherichia coli*, *Pseudomonas aeruginosa*, *Candida albicans, Xanthomonas campestris *pv.* vesicatoria*, *Pseudomonas syringae *pv.* phaseolicola*, *Enterobacter cloacae*, and *Humulus lupulus *[91, 112–119]. However, *Scaligeria tripartite *essential oils can be detected with *Colletotrichum acutatum* [120]. *Erythrina vogelii *root was visualized with *Cladosporium cucumerinum *[121]. *Gladiolus dalenii *van Geel (Iridaceae) bulb extracts were detected with *Aspergillus niger* [122]. The antibacterial compounds from extract from *Urginea sanguinea *bulbs were visualized with *Staphylococcus aureus *[123]. The rapid TLC autobiographic methods were also elaborated for the detection of acetylcholinesterase and butyrylcholinesterase inhibitors in various plants [124, 125].

## 4. Conclusion

- In this review, known indicators and dyes applied as new visualizing reagents and various visualizing systems as well as photocatalytic reactions and microbiological detection (bioautography detection) described should serve as a supplement to those used previously for the detection of selected drugs.
- The visualizing effect depends on the chemical structure of the visualizing reagent, the structure of the substance detected, and the chromatographic adsorbent applied.
- Particular application will have those visualizing reagents, which, with substances present in analyzed mixtures, will give diversified colors of chromatographic spots.
- The visualizing results of detected drugs depend on method of application—spraying or dipping.
- Broadening index, detection index, characteristics of densitometric band, modified contrast index, limit of detection, densitometric visualizing index, and linearity range of detected compounds can be used for the evaluation of visualizing effects of applied visualizing reagents.
- Presented data indicate the detection progress of selected drugs investigated by thin-layer chromatography.

## Figures and Tables

**Table 1:** Fundamental absorption band of drugs, color of spot of detected drugs, background color of chromatogram, limit of detection, and linearity range.

Detected drug	Stationary phase	Detection way	Fundamental absorption band of drug *λ*_max_ (nm)	Color of spot of detected drug	Background color of chromatogram	Limit of detection (*μ*g)	Linearity range (*μ*g spot^−1^) (*r*, correlation coefficient)	Reference
Estradiol	Neutral aluminum oxide 60F_254_ toluene + ethyl acetate (1 : 1, v/v)	Without using visualizing reagent	205	Lack of colored spot in visible light	White	0.32	1.10 ÷ 25.00 (*r* = 0.9951)	[43]
Estradiol		Barton's reagent	233	Dark blue/green	Willow-green	0.32	0.53 ÷ 10.24 (*r* = 0.9975)	[43]
Estradiol		Sulphuric acid	278	Light orange	Light beige	0.10	0.88 ÷ 12.80 (*r* = 0.9974)	[43]
Estradiol		Gentian violet	226	Violet/blue	Violet	0.32	0.53 ÷ 25.00 (*r* = 0.9943)	[43]
Estradiol		Methylene violet	227	Grey/blue	Light grey	0.32	0.88 ÷ 3.36 (*r* = 0.9975)	[43]

Estradiol	Neutral aluminum oxide 150F_254_ toluene + ethyl acetate (1 : 1, v/v)	Without using visualizing reagent	207	Lack of colored spot in visible light	White	0.32	1.37 ÷ 20.00 (*r* = 0.9972)	[43]
Estradiol		Gentian violet	598	Dark violet	Violet	0.32	0.88 ÷ 20.00 (*r* = 0.9879)	[43]
Estradiol		Methylene violet	204	Grey/blue	Light grey	0.53	1.37 ÷ 25.00 (*r* = 0.9983)	[43]

Salicylanilide	Silica gel 60F_254_ (#1.05744) chloroform	Without using visualizing reagent	307	Lack of colored spot in visible light	White	0.07	5.00 ÷ 30.00 (*r* = 0.9802)	[44]
Salicylanilide		Brilliant green	597	Dark green	Green	0.70	3.00 ÷ 30.00 (*r* = 0.9929)	[44]

(±)-*α*-Tocopherol	Silica gel 60 toluene	Without using visualizing reagent	270	Lack of colored spot in visible light	White	0.45	3.46 ÷ 20.00 (*r* = 0.9894)	[41]
(±)-*α*-Tocopherol		2,2′-Bipyridine-iron (III) chloride	524	Raspberry red	Light beige	2.08	5.76 ÷ 30.00 (*r* = 0.9927)	[41]
(±)-*α*-Tocopherol		Methylene violet	270	Azure with white border	Light grey-blue	0.30	0.75 ÷ 5.76 (*r* = 0.9949)	[41]
(±)-*α*-Tocopherol		Methyl green	270	Light green	Green	0.30	0.75 ÷ 5.76 (*r* = 0.9863)	[41]

(+)-*α*-Tocopherol acetate	Silica gel 60 toluene	Without using visualizing reagent	203	Lack of colored spot in visible light	White	0.30	0.75 ÷ 20.00 (*r* = 0.9978)	[41]
(+)-*α*-Tocopherol acetate		Rhodamine B	203	Dark pink	Pink	0.45	1.25 ÷ 16.00 (*r* = 0.9936)	[41]
(+)-*α*-Tocopherol acetate		Gentian violet	203	Dark violet	Violet	0.75	2.08 ÷ 25.00 (*r* = 0.9908)	[41]
(+)-*α*-Tocopherol acetate		Methyl green	203	Green (small contrast with background color)	Green	0.75	1.25 ÷ 25.00 (*r* = 0.9943)	[41]
(+)-*α*-Tocopherol acetate		Janus blue	203	Dark blue	Blue	0.75	1.25 ÷ 25.00 (*r* = 0.9938)	[41]

Ibuprofen	Silica gel 60F_254_ chloroform + methanol (50 : 1.4, v/v)	Without using visualizing reagent	200	Lack of colored spot in visible light	White	0.62	1.25 ÷ 10.00 (*r* = 0.9958)	[42]
Ibuprofen		Methylene violet	200	White-blue with dark blue border	Light	1.25	5.00 ÷ 25.00 (*r* = 0.99549)	[42]

Stearic acid	Silica gel 60 n-hexane + acetone (4 : 1, v/v)	Rhodamine B	585	Dark pink	Pink	1.25	1.56 ÷ 28.40 (*r* = 0.9913)	[45]
Stearic acid		Methylene blue	687	Blue with light border	Blue	0.07	0.41 ÷ 7.44 (*r* = 0.9975)	[45]
Stearic acid		Janus blue	617	Navy blue with white border	Blue	0.12	1.56 ÷ 7.44 (*r* = 0.9928)	[45]

Stearyl alcohol	Silica gel 60 n-hexane + ethyl acetate + methanol (46 : 3.5 : 0.5, v/v/v)	Rhodamine B	586	Dark pink	Pink	1.48	2.90 ÷ 27.00 (*r* = 0.9964)	[45]

Stearyl alcohol	Silica gel 60 n-hexane + ethyl acetate + methanol (46 : 3.5 : 0.5, v/v/v)	Malachite green	644	Green with light border	Green	0.32	0.89 ÷ 8.85 (*r* = 0.9959)	[45]

Stearyl alcohol	Silica gel 60 n-hexane + ethyl acetate + methanol (46 : 3.5 : 0.5, v/v/v)	Janus blue	655	Navy blue	Blue	0.32	0.53 ÷ 7.08 (*r* = 0.9995)	[45]

**Table 2:** Characteristics of densitometric band of detected drugs, modified contrast index, densitometric visualizing index, modified broadening index, and detection index.

Detected drug	Stationary phase, mobile phase	Detection way	Densitometric band characteristic of detected drug			Modified contrast index (AU/°)	Densitometric visualizing index (Au/*μ*g·°)	Modified broadening index (*μ*g/AU)	Detection index (*μ*g/AU)	Reference
			Area (AU)	Height (AU)	*α* (°)					
Estradiol	Neutral aluminum oxide 60F_254_ toluene + ethyl acetate (1 : 1, v/v)	Without using visualizing reagent	13048	246	14.5	16.966	—	1.916	0.32/495	[43]
Estradiol		Barton's reagent	23786	255	13	19.615	—	1.051	0.32/1070	[43]
Estradiol		Sulphuric acid	44709	497	13	38.231	—	0.559	0.10/850	[43]
Estradiol		Gentian violet	11348	220	15	14.667	—	2.203	0.32/760	[43]
Estradiol		Methylene violet	13622	221	17	13.000	—	1.835	0.32/705	[43]

Estradiol	Neutral aluminum oxide 150F_254_ toluene + ethyl acetate (1 : 1, v/v)	Without using visualizing reagent	11424	192	13	14.769	—	2.188	0.32/409	[43]
Estradiol		Gentian violet	10906	127	20	7.700	—	2.292	0.32/796	[43]
Estradiol		Methylene violet	8466	154	33	4.667	—	2.953	0.53/490	[43]

Salicylanilide	Silica gel 60F_254_ (#1.05744) chloroform	Without using visualizing reagent	72889	707	9	78.5	11.570	0.686	0.07/1590	[44]
Salicylanilide		Brilliant green	91842	704	8	88.0	1.640	0.544	0.70/11112	[44]

(±)-*α*-Tocopherol	Silica gel 60 toluene	Without using visualizing reagent	34519	459	16	28.69	0.479	0.724	0.45/3100	[41]
(±)-*α*-Tocopherol		2,2′-Bipyridine-iron (III) chloride	60474	698	9	77.56	0.323	0.413	2.08/8915	[41]
(±)-*α*-Tocopherol		Methylene violet	40426	636	8	79.50	1.684	0.618	0.30/3456	[41]
(±)-*α*-Tocopherol		Methyl green	37196	576	9	64.00	1.378	0.672	0.30/1564	[41]

(+)-*α*-Tocopherol acetate	Silica gel 60 toluene	Without using visualizing reagent	33796	591	11	53.73	1.024	0.740	0.30/1100	[41]
(+)-*α*-Tocopherol acetate		Rhodamine B	26031	455	15	30.33	0.386	0.960	0.45/1511	[41]
(+)-*α*-Tocopherol acetate		Gentian violet	29441	545	10	54.50	0.392	0.849	0.75/1884	[41]
(+)-*α*-Tocopherol acetate		Methyl green	30226	564	11	51.27	0.366	0.827	0.75/3911	[41]
(+)-*α*-Tocopherol acetate		Janus blue	30125	565	11	51.36	0.365	0.830	0.75/3750	[41]

Ibuprofen	Silica gel 60F_254_	Without using visualizing reagent	55644	445	26	17.115	0.342	0.449	0.62/3325	[42]
Ibuprofen	chloroform + methanol (50 : 1.4, v/v)	Methylene violet	53696	348	35	9.943	0.123	0.466	1.25/4438	[42]

Stearic acid	Silica gel 60 n-hexane + acetone (4 : 1, v/v)	Rhodamine B	6567	82	31	2.645	0.017	4.32	1.25/900	[45]
Stearic acid		Methylene blue	30918	368	14	26.286	3.155	0.92	0.07/245	[45]
Stearic acid		Janus blue	36175	462	11	42.000	2.741	0.79	0.12/604	[45]

Stearyl alcohol	Silica gel 60 n-hexane + ethyl acetate + methanol (46 : 3.5 : 0.5, v/v/v)	Rhodamine B	3605	100	13	7.692	0.019	7.49	1.48/234	[45]
Stearyl alcohol		Malachite green	12378	333	7	47.571	0.553	2.18	0.32/382	[45]
Stearyl alcohol		Janus blue	10963	269	13	20.692	0.264	2.46	0.32/214	[45]

**Table 3:** Detection results of selected corticosteroids [46].

Corticosteroid	*R*_*F*_	Reagent 1		Reagent 2	
		Color of corticosteroid spot	Limit of detection (*µ*g)	Color of corticosteroid spot	Limit of detection (*µ*g)
Prednisolone	0.40	Blue-green	0.400	Violet	0.150
Dexamethasone	0.45	Bray-violet	0.300	Violet	0.100
Triamcinolone	0.29	Grayish	0.200	Violet	0.075
Beclomethasone	0.38	Blue	0.060	Violet	0.040
Prednisone	0.50	Brown-yellow	0.300	Violet	0.100
Betamethasone	0.38	Blue	0.050	Violet	0.030
Hydrocortisone	0.39	Greenish	0.050	Violet	0.040

**Table 4:** Characteristics of densitometric bands of stigmasterol after dipping and spraying with the use of particular visualizing reagents [47].

	10 *µ*g of stigmasterol				5 *µ*g of stigmasterol			
	Area (AU)	Height (AU)	Angle *α* (°)	*λ*_max_ (nm)	Area (AU)	Height (AU)	Angle *α* (°)	*λ*_max_ (nm)
Without using visualizing reagent	7111	198	9	200	4400	126	9	200
Rhodamine B								
Dipping	2264	86	32	200	1040	37.3	12	591
Spraying	4753	143.3	9	200	2721	90.6	14	200
Gentian violet								
Dipping	2685	95.8	76	200	712	30.1	34	693
Spraying	5844	179.5	7.5	601	4655	137.7	8	602
Methylene violet								
Dipping	3792	119	13.5	200	1177	58.2	12	200
Spraying	8988	251.2	7	200	6122	176.6	8	200
Methyl green								
Dipping	3514	93.4	73	200	2237	55.7	39	200
Spraying	7857	216	7	200	5447	158.3	12	200
Malachite green								
Dipping	2350	84.4	73.5	200	1052	37.3	37	200
Spraying	7740	221	10	638	4942	151.6	13	636
Janus blue								
Dipping	2334	82.6	66	200	1429	47.5	36	663
Spraying	4024	147.3	9.5	674	2302	86.2	11.5	674

**Table 5:** Characteristics of densitometric bands of *β*-sitosterol after dipping and spraying with the use of particular visualizing reagents [47].

	10 *µ*g of *β*-sitosterol				5 *µ*g of *β*-sitosterol			
	Area (AU)	Height (AU)	Angle *α* (°)	*λ*_max_ (nm)	Area (AU)	Height (AU)	Angle *α* (°)	*λ*_max_ (nm)
Without using visualizing reagent	4797	144.5	15	200	3243	82.6	10	200
Rhodamine B								
Dipping	646	34	110	200	112	9.4	21	595
Spraying	3807	115.8	12	200	1770	59.6	22.5	584
Gentian violet								
Dipping	7161	312.3	45	510	1418	47.8	5	517
Spraying	5148	146.9	9.5	200	4354	112.4	19	606
Methylene violet								
Dipping	3017	110	19	200	1490	49	9.5	200
Spraying	7561	213.9	8.5	200	4056	126.3	10	200
Methyl green								
Dipping	1415	53.3	105	200	538	24.8	47	473
Spraying	5966	167.4	9	200	4560	103.5	16	200
Malachite green								
Dipping	2235	82.7	51	640	966	40.7	42	643
Spraying	5338	155.5	8	643	3444	111.4	10	649
Janus blue								
Dipping	1029	49	134	678	53	6.2	41.5	685
Spraying	2474	69.1	24	667	1772	50.1	30	662

**Table 6:** Characteristics of the visualizing effect for selected phenolic compounds.

Detected drug	Stationary phase	Mobile phase	Visualizing reagent	Detection limit (*µ*g)	Detectability index (*µ*g/mm^2^)	Color of spot	Background color of chromatogram	Reference
Adrenaline	Silica gel 60F_254_	Glacial acetic acid + n*-*butanol + water (1 : 4 : 1, v/v/v)	Brilliant cresyl blue in 5% NaOH	0.10	0.10/10	Brown	Light violet	[61]
	Silica gel 60/kieselguhr F_254_		Bromocresol green in 5% NaOH	0.25	0.25/47	Light brown	Sea/green	
	Polyamide 11F_254_		Eosin yellow in 5% NaOH	2.50	2.50/30	Light brown	Light yellow	

Dopamine	Silica gel 60F_254_	Glacial acetic acid + n*-*butanol + water (1 : 4 : 1, v/v/v)	Bromocresol green in 5% NaOH	0.10	0.10/15	Orange	Light blue	[61]
	Silica gel 60/kieselguhr F_254_			0.25	0.25/10	Brown/orange	Sea/green	
	Polyamide 11F_254_		Eosin yellow in 5% NaOH	0.75	0.75/17	Light brown	Light yellow	

Metaraminol	Silica gel 60F_254_	Glacial acetic acid + n*-*butanol + water (1 : 4 : 1, v/v/v)	Bromocresol green in 5% NaOH	0.50	0.50/15	Lemon	Light blue	[61]
	Silica gel 60/kieselguhr F_254_		Eriochrome black T in 5% NaOH	0.50	0.50/35	Brown	Light brown	
	Polyamide 11F_254_		Aniline blue in water	50	50/42	Light yellow	Blue	

Fenoterol	Silica gel 60F_254_	Glacial acetic acid + n*-*butanol + water (1 : 4 : 1, v/v/v)	Bromocresol green in 5% NaOH	0.10	0.10/13	Brown/orange	Light blue	[61]
	Silica gel 60/kieselguhr F_254_		Eriochrome black T in 5% NaOH	0.50	0.50/38	Brown	Light brown	
	Polyamide 11F_254_		Eosin yellow in 5% NaOH	5.0	5/15	Yellow	Light yellow	

Phenylephrine	Silica gel 60F_254_	Glacial acetic acid + n*-*butanol + water (1 : 4 : 1, v/v/v)	Bromocresol green in 5% NaOH	0.25	0.25/21	Lemon	Light blue	[61]
	Silica gel 60/kieselguhr F_254_		Eriochrome black T in 5% NaOH	0.50	0.50/41	Brown	Light brown	

Bithionol	Silica gel 60F_254_	Glacial acetic acid + n*-*butanol + water (1 : 4 : 1, v/v/v)	Brilliant cresyl blue in 5% NaOH	0.50	0.50/77	Brown	Light violet	[61]
	Silica gel 60/kieselguhr F_254_		Brilliant cresyl blue in 5% NaOH	2.50	2.50/68	Brown	Light brown	

Methyldopa	Silica gel 60	Glacial acetic acid + n*-*butanol + water (1 : 4 : 1, v/v/v)	Thymol blue in 2% NaOH	3.2	3.2/7	Brown/black	Beige	[62]
Norepinephrine			Bromothymol blue in 2% NaOH	0.6	0.6/28	Yellow/brown	Light blue	
Terbutaline			Brilliant cresyl blue in 2% NaOH	2.0	2.0/17	Light brown	Light blue	
Bamethane			Aniline blue in 2% NaOH	10	10/25	Blue	Light blue	
Etamivan			Aniline blue in 2% NaOH	50	50/18	White/blue	Light blue	

Phloroglucinol	Silica gel 60	Chloroform + methanol (9 : 1, v/v)	Brilliant cresyl blue in 2% NaOH	2.0	2.0/23	Light brown	Light blue	[62]
Pentazocine			Aniline blue in 2% NaOH	0.5	0.5/68	White	Light blue	
Hexachlorophene			Thymol blue in 2% NaOH	5.0	5.0/37	Yellow/beige	Beige	
Pyrocatechine			Brilliant green in 2% NaOH	0.3	0.3/17	Light orange/red	Light green	
Niclosamide			Brilliant green in 2% NaOH	0.8	0.8/18	Green	Light green	
Salicylanilide			Brilliant green in 2% NaOH	0.8	0.8/16	Green	Light green	
Thymol			Brilliant green in 2% NaOH	2.0	2.0/42	Yellow green	Light green	

Eugenol	Silica gel 60	Benzene	Bromothymol blue in 2% NaOH	5.0	5.0/37	Light yellow	Light blue	[62]
